# Drug anaphylaxis in children – data of the German-speaking anaphylaxis registry

**DOI:** 10.1186/2045-7022-3-S3-O2

**Published:** 2013-07-25

**Authors:** S Gernert, M Worm, H Ott, S Hompes, L Lange

**Affiliations:** 1 Department of Pediatrics, St. Marien-Hospital Bonn, Bonn, Germany; 2 Department of Dermatology and Allergy, Charité – Universitätsmedizin Berlin, Berlin, Germany; 3 Department of Pediatric Dermatology, Catholic Children's Hospital Wilhelmstift Hamburg, Hamburg, Germany

## Background

Anaphylaxis caused by drugs seems to be less common in children compared with adults. The anaphylaxis-registry collects data of patients with severe allergic reactions from 89 allergy centres in Germany, Austria and Switzerland. Aim of this study was to investigate the circumstances and relevance of drug-induced anaphylaxis in children.

## Methods

The analysed data included anaphylactic reactions registered from July 2006 until February 2012.

The data are delivered by a password-controlled internet-based-questionnaire. Only severe reactions with pulmonary and/or cardiovascular symptoms are accepted. 704 of the 2926 anaphylactic reactions occurred in children. They were caused by foods (406 cases), insects (180 cases), drugs (50 cases) and others (68 cases).

## Results

19 of 50 cases of drug induced anaphylaxis were induced by subcutaneous immunotherapy (SCIT). In the 31 remaining cases most frequent triggers were analgetics (n=14) and antibiotics (n=10) followed by a small number of other drugs such as fentanyl and lidocaine. Nearly all of the children suffered from respiratory symptoms (86%) in addition to skin symptoms, 50% had cardiovascular symptoms and only a small number (22%) had gastrointestinal symptoms. Most of the anaphylactic reactions after SCIT started at the location (medical center, hospital) where the SCIT was administered; only 3 of the SCIT-induced reactions started after the patients left the medical center. Most of the anaphylactic reactions triggered by other drugs took place at home. Only 35% occurred in a medical center or hospital.

Although only severe reactions were included, only a small number of the children (12%, 42% of the reactions related to SCIT) received intramuscular, intravenous or inhalative adrenaline. Most of the children (67%, 84%) were treated with antihistamines and/or corticosteroids. Inhaled beta-2-agonists were part of the anaphylaxis therapy in only 19% (SCIT 42%).

38% of the children received an emergency kit after the anaphylactic reaction. This kit included self-injectable adrenaline in 20%, antihistamine in 32%, inhaled beta-2-agonists in 14% and corticosteroids in 36%.

## Conclusion

Drug-induced anaphylaxis in children is mainly triggered by analgetics, antibiotics and SCIT. Most of the reactions take place at the children’s home and not in hospitals or medical centers.

## Disclosure of interest

None declared.

